# The Effect of Different Luteal Phase Support Applications on Clinical Pregnancy Outcomes in Frozen-Thawed Embryo Transfer

**DOI:** 10.1155/2023/8157210

**Published:** 2023-07-24

**Authors:** Elif Ganime Aygün, Esra Özbaşlı, Mehmet Faruk Köse

**Affiliations:** ^1^Atakent Hospital, Department of Gynecology and Obstetrics, Acıbadem Mehmet Ali Aydınlar University, Istanbul, Turkey; ^2^School of Medicine, Department of Gynecology and Obstetrics, Acıbadem Mehmet Ali Aydınlar University, Istanbul, Turkey

## Abstract

**Purpose:**

During the frozen-thawed embryo transfer (FET) method, controlled ovarian hyperstimulation is used. At the same time, progesterone support is given for luteal phase support. In this study, we investigated the effects of various luteal phase support agents administered orally, intramuscularly (IM), and vaginally during FET on pregnancy rates.

**Methods:**

The files of 166 patients between the ages of 21 and 44 in the Assisted Reproductive Techniques Center of Acıbadem Mehmet Ali Aydınlar University Atakent Hospital were analyzed retrospectively between 2016 and 2022. The patients' FSH, LH, E2, P4, AMH, and TSH levels were measured. The GnRH antagonist protocol was initiated on the 2nd or 3rd day of menstruation. Three types of progesterone agents were used in females with PCOS. Three different methods were applied: 50 mg/ml of IM progesterone daily, 90 mg of progesterone gel 2∗1 vaginally, and dydrogesterone acetate tb. orally 3∗1. FET was performed on women who received 21 days of treatment by thawing 5th-day embryos. B-hCG was performed on the 12th day after the transfer, and evaluations were made. The study results were evaluated as follows: for the whole study group, for those <30 years of age, for those 30-35 years of age, and for those >35 years of age.

**Results:**

A total of 164 patients, 57 females using vaginal progesterone gel, 30 females using oral progesterone tablet, and 77 females using IM progesterone, who met the inclusion criteria, were included in the study. The pregnancy outcomes of IM progesterone application were statistically significantly higher in the entire study group and the >35 age group when compared to the vaginal progesterone gel application. It was found that the pregnancy outcomes of IM progesterone application increased statistically significantly in the <30 age group when compared to outcomes in the other groups, using vaginal progesterone gel and oral progesterone tb.

**Conclusions:**

We found that IM progesterone application was more effective than vaginal progesterone gel application for luteal phase support. Many randomized controlled, especially live birth rate studies, are required before results can more closely approximate those for the general population.

## 1. Introduction

Frozen-thawed embryo transfer (FET) is preferred over fresh IVF due to the lower risk of ovarian hyperstimulation syndrome [1]. The majority of the studies on FET have focused on “freezing and thawing” lab techniques [2]. In addition, it is critical to perform embryo transfer (ET) after the endometrium and the implantation tissue have reached optimal conditions with the help of the luteal phase. Many studies on the use of progesterone by vaginal, oral, and intramuscular (IM) routes, alone or in combination with HRT, have been conducted [3, 4]. Oligoovulatory females with ovarian dysfunction, in particular, were seen to benefit significantly [5].

Although progesterone taken orally has a positive effect, it is believed that a high dose is required to achieve effective blood concentrations due to the first-pass effect through the liver. There are also systemic side effects. It is possible to obtain effective blood concentrations easily in IM applications. However, injection necessitates medical personnel, and injections carry risks. Vaginal progesterone, on the other hand, acts directly on the uterus and prepares the endometrium. The main point of concern in relation to the use of vaginal progesterone is whether it is sufficiently absorbed from the vaginal tissue [6].

In this study, we investigated the effects of various luteal phase support agents administered orally, IM, and vaginally during FET on pregnancy rates. Our limitation in this study is that only clinical pregnancy rates were determinatem, and live birth rates were not given. In addition, the other limitation is that subcutaneous progesterone agents have not been compared. We also evaluated endometrial thickness, BMI, and serum estradiol (E2) and progesterone (P4) levels on transfer day.

## 2. Materials and Methods

### 2.1. Ethics

The study was accomplished following ethical principles according to the Declaration of Helsinki 1964. This retrospective study was approved by the Ethics Committee of the Acıbadem Mehmet Ali Aydınlar University, School of Medicine.

### 2.2. Data Collection and Patient Selection

The study was conducted by retrospectively analyzing the files of 166 patients between the ages of 21 and 44 at the Assisted Reproductive Techniques Center of the Acıbadem Mehmet Ali Aydınlar University Atakent Hospital between 2016 and 2022. The patients' FSH, LH, E2, P4, AMH, and TSH levels were measured. The GnRH antagonist protocol was initiated on either the 2nd or 3rd day of menstruation. The patients were called to the outpatient clinic at an interval of 3-4 days. The patients' blood E2 levels were monitored. After the follicles, which reached 17 mm in size, were cracked with the ovulation induction agent Ovitrelle 250 micrograms/0.5 ml (Merck Serono S.p.A., Bari, Italy). Oocyte collection was performed under general anesthesia. All embryos developed in vitro for 5 days were frozen. Then, estrogen, which was initiated on the 2nd day of the menstrual period, was given to all patients as 2 mg 3∗1 (Estrofem tb., Novo Nordisk Sağlık Ürünleri Tic. Ltd. Şti. İstanbul, Türkiye). Apart from the basal E2 hormone, the patients' weekly E2 values were also measured. Transvaginal ultrasonography was also used to measure endometrial thickness. After 15 days of estrofem treatment, 3 types of progesterone agents were administered to the patients. Progesterone (Progestan 50 mg/ml, Koçak Farma İlaç ve Kimya Sanayi A.Ş. İstanbul, Türkiye), one of which is natural micronized progesterone, was administered daily IM. The other method was vaginal; 90 mg of progesterone gel (Crinone 8% vaginal gel, İlaç Ecza ve Kimya Tic.A.Ş., İstanbul, Türkiye) was applied vaginally at a dose of 2^∗^1. Finally, dydrogesterone acetate (Duphaston tb., Abbott Laboratories İth. İhr.ve Tic. Ltd. Şti, İstanbul, Türkiye) was applied orally as 3∗1. FET was performed on women, who received 21 days of treatment by thawing 5th-day embryos. The B-hCG was performed on the 12th day after the transfer, and evaluations were made. Inclusion criteria were being primarily infertile, being between the ages of 21 and 44, having undergone the FET protocol, and having a BMI between 18 and 40 kg/m^2^. The exclusion criteria were as follows: a live birth history (secondary infertile couple), a history of chemoradiotherapy, the presence of a chronic illness, substance abuse, and severe ovarian failure.

### 2.3. Statistical Analysis

In the analysis of the variables, SPSS 25.0 (IBM Corporation, Armonk, New York, United States), PAST 3 (Hammer, Ø., Harper, D.A.T., Ryan, P.D. 2001, Paleontological Statistics), and Medcalc 14 (Acacialaan 22, B-8400 Ostend, Belgium) programs were used. The conformity of univariate data to normal distribution was evaluated with the Shapiro-Wilk-Francia test, while the homogeneity of variance was evaluated with the Levene test. While the Mardia (Dornik and Hansen Omnibus) test was used for the conformity of multivariate data to the normal distribution, the Box-M test was used for variance homogeneity. In order to compare more than two independent groups with each other based on quantitative variables, the one-way ANOVA test was used with the Bootstrap test, and the Kruskal-Wallis H test was used in conjunction with the Monte Carlo simulation; Dunn's test was used in post hoc analyses. The Wilcoxon test (Monte Carlo) was used to compare dependent quantitative variables with two repeated measurements. In the comparison of categorical variables with each other, the Fisher-Freeman-Halton and Pearson chi-square tests were tested using the Monte Carlo simulation technique. The Backward method was used in conjunction with the logistic regression test to determine the cause-effect relationship of the groups with the explanatory variables. Quantitative variables were expressed as mean (standard deviation) and median (minimum/maximum) in the tables, while categorical variables were shown as n (%). The variables were analyzed at a 95% confidence level, and a *p* value of less than 0.05 was considered significant. Based on the statistical comparison results made according to reference publications, the number of samples was calculated as follows: *n* = 68 (Wanggren et al., 2022). Type 1 error was determined as 5%, and the power of the study was 80%.

Power analysis G power version 3.1 of our study was used for all included patients. For the logistic regression with a sample size of 164 observations, *Pr* (*Y* = 1|*X* = 1) H0 = 0.33, our smallest odd ratio was calculated as 1.74, and the power at the 0.05 significance level was 94%.

## 3. Results

A total of 164 patients, 57 females using vaginal progesterone gel, 30 females using oral progesterone tablet, and 77 females using IM progesterone, who met the inclusion criteria, were included in the study.  Table 1 shows the age, BMI, infertility reasons, pregnancy rates, endometrial echogenicity thicknesses, IVF trials, serum FSH, LH, AMH, TSH, basal and transfer days E2, and P4 levels of the entire study group and females, who received different luteal phase support agents.

The pregnancy rate and serum LH levels differed statistically significantly between groups based on the factors assessed. Pregnancy was observed at a rate of 66.2% in the P4 IM group, 56.7% in the oral progesterone group, and 42.1% in the vaginal progesterone gel group. When compared to the vaginal progesterone group, the progesterone IM group had a statistically significant higher rate. In the evaluation of serum LH levels, the median values were found to be 5.21 IU/l in the IM progesterone group, 6.72 IU/l in the oral progesterone group, and 5 IU/l in the vaginal progesterone group (Table 2).

Age, BMI, infertility causes, pregnancy rates, endometrial echogenicity thicknesses, IVF trials, serum FSH, LH, AMH, TSH, basal and transfer days E2, and P4 levels for different luteal phase support agents for the group <30 years of age are specified in Table 3. The pregnancy rate was found to be statistically significantly different based on these factors. Pregnancy rates in the IM progesterone group, the vaginal progesterone group, and the oral progesterone group were found to be 88.2%, 50%, and 33.3%, respectively. It was observed that the pregnancy rates of the IM progesterone group were statistically significantly higher than those of the other groups.

Age, BMI, infertility causes, pregnancy rates, endometrial echogenicity thicknesses, IVF trials, serum FSH, LH, AMH, TSH, basal and transfer days E2, and P4 levels for different luteal phase support agents for the group 30-35 years of age are specified in Table 4. The age factor was found to be statistically significantly different based on these factors. The median age was 32 years in the IM progesterone group, 31 in the vaginal progesterone group, and 32 in the oral progesterone group. A statistically significant difference was detected between the vaginal progesterone-oral progesterone groups. In terms of basal P4 levels, the value for the IM progesterone group was found to be 0.76 ng/ml and the value for the vaginal progesterone group to be 0.52 ng/ml. A statistically significant difference was found between the groups.

Age, BMI, infertility causes, pregnancy rates, endometrial echogenicity thicknesses, IVF trials, serum FSH, LH, AMH, TSH, basal and transfer days E2, and P4 levels for different luteal phase support agents for the group <35 years of age are specified in Table 5. According to these factors, the IM progesterone and oral progesterone groups demonstrated a statistically significant increase in serum P4 levels on the day of transfer when compared to those in the vaginal progesterone group. When the pregnancy rates were compared, the rates were found to be 58.1% in the IM progesterone group, 46.2% in the oral progesterone group, and 20% in the vaginal progesterone group. It was observed that there was a statistically significant difference between the IM progesterone group and the vaginal progesterone group.

In order to better understand the pregnancy outcomes, the relationship between age, treatment agents used, FSH, transfer day progesterone values, and pregnancy outcomes was evaluated (Table 6). A negative correlation was found between FSH and age and pregnancy outcome (OR, respectively, 1.12 (1.01-1.25), 1.08 (1.01-1.16)). A positive correlation was found between the progesterone values on the transfer day and the pregnancy outcome. In a comparison of pregnancy outcomes with respect to the use of vaginal progesterone gel and oral progesterone tb, it was found that the pregnancy rate increased for oral progesterone tb (OR 3.66 (1.61-8.31)).

In multiple logistic regression analysis, a difference in the pregnancy success rate was found between oral progesterone tb and vaginal progesterone gel applications in females aged >35 years (OR 7.29 (1.58-33.58)). A difference was found between the serum progesterone level on the transfer day and the basal serum progesterone level in females 30-35 years of age (OR 1.06 (1.01-1.11)) (Table 7).

## 4. Discussion

Estrogen applications started in the early follicular phase suppress follicle selection and the LH peak, preventing ovulation. However, the serum LH level is crucial for endometrial receptivity [7]. In their study, Khoury et al. divided the females, who received artificial FET into two groups: the group with doubled LH levels and the group with unchanged LH levels [8]. Serum E2 levels and P4 levels were higher in the group with doubled LH compared to those in the group with unchanged LH. However, no statistically significant difference was observed in terms of clinical pregnancy, live birth rate, abortion, and ectopic pregnancy. In their study, Griesinger et al. found no correlation between serum LH levels and pregnancy outcomes in transdermal E2 patch applications [9]. In our study, the serum LH level was observed to be statistically significantly elevated in females, who had received oral progesterone tb. However, the pregnancy rate was found to be statistically significantly higher in the progesterone IM group.

Serum AMH, antral follicle count, and serum FSH measurement between the 2nd and 5th days of menstruation are crucial for ovarian reserve evaluation [10]. Therefore, it is also a marker for the number of oocytes to be obtained in IVF applications. In their study, Çakıroğlu et al. divided females, who had undergone IVF with controlled ovarian hyperstimulation into 4 groups as follows: normal-weight and obese females, and females with and without PCOS [11]. Among normal-weight females, there was no statistically significant difference in serum FSH levels between groups; however, serum FSH levels were statistically higher in the obese PCOS group compared to those in the obese non-PCOS group. When serum AMH levels were compared, serum AMH levels were found to be higher in both groups of females with PCOS. However, no statistically significant difference was observed in terms of pregnancy outcomes. It was observed that there was no statistically significant difference in serum FSH and AMH levels between all groups in our study. This is a powerful case for a comparison of the different administration routes of progesterone agents, as undertaken in our study.

The luteal phase of menstruation is the phase that occurs after the ovulation phase and is responsible for the formation of the corpus luteum through progesterone. It is significant for implantation. For this reason, the serum P4 level and progesterone support, if necessary, are important in patients, who will undergo FET [12]. Volovsky et al. evaluated the pregnancy outcomes and the serum P4 level on the FET day [13]. Based on the serum P4 levels, 5 ng/ml, 10 ng/dl and 20 ng/dl values were accepted as cut-off values, and the examination was performed. No statistically significant difference was observed in the 10 ng/dl and 20 ng/dl groups in terms of pregnancy rate, clinical pregnancy rate, and live birth rate, irrespective of whether these values were less or more. When the cut-off value was accepted as 5 ng/dl, while no statistically significant difference was detected in biochemical and clinical pregnancy rates, a significant difference was detected in live birth rates. In the study of Boynukalın et al., females who underwent FET were divided into two groups according to the following: the presence of an ongoing pregnancy and the absence of an ongoing pregnancy [14]. In the ongoing pregnancy group, the serum P4 level was found to be statistically significantly higher compared to that in the other group. In the ROC analysis, the cut-off value for the continuation of pregnancy was found to be 20.6 ng/ml. Although there are studies in the literature that measure serum P4 on the day of the pregnancy test [15], we only measured baseline P4 and transfer day P4 since we believed that measuring serum P4 on the day of the pregnancy test would be inconsistent. In our study, when the entire study group was examined, no statistically significant difference was found in serum P4 examination on the day of transfer. When the age groups were examined, in the group aged >35 years, the serum P4 level of females, who received IM progesterone and used oral progesterone, was found to be statistically significantly higher than that of the group using vaginal progesterone gel. Alvarez et al., on the other hand, approached from a different angle, stating that luteal phase support should be individualized, and they measured the females' serum P4 values one day before the FET. The patients with serum P4 levels >10.6 ng/ml underwent FET as standard the next day. The patients with serum P4<10.6 ng/ml were administered progesterone by subcutaneous (SC) injection. After administration, 98.2% of females had a serum P4 level of >10.6 ng/ml. When pregnancy outcomes were compared, no statistically significant difference was observed between clinical pregnancy, ongoing pregnancy, live birth, and miscarriage rates [6].

Wang et al. applied vaginal progesterone gel (90 mg) or IM progesterone (40 mg/day) for luteal phase support to females, who had received oral dydrogesterone (20 mg/day) and estradiol valerate (4–8 mg/day) [16]. In terms of live birth, clinical pregnancy, implantation, miscarriage, and ectopic pregnancy, no statistically significant difference was found between the groups. The results of this study indicate that progesterone in the form of a gel applied vaginally may be a good alternative to progesterone administered by the IM route. The difference between this study and ours is that all females were given oral dydrogesterone in the aforementioned study. In their study, Shapiro et al. compared vaginal progesterone gel (90 mg/day) application and IM progesterone (50 mg/day) injection in those who had undergone FET [17]. The results of this study demonstrated that there was no statistically significant difference between the groups in terms of implantation rate, clinical pregnancy rate, or live birth rate. In the study by Klement et al., the groups that received a 200 mg vaginal progesterone injection (three times daily) and a 50 mg IM progesterone injection were compared [18]. Serum P4 concentration in the group, which had received IM progesterone was statistically significantly higher compared to that in the group, which had received vaginal progesterone. One day before the transfer day, serum P4 levels increased significantly in the IM progesterone group. However, this difference did not affect subendometrial contractility. In our study, 3 different groups, including the oral dydrogesterone group, were compared. The pregnancy rate in the IM progesterone group was seen to be higher than that of the vaginal progesterone group. When the age groups were examined and the pregnancy rates were compared to those in the group aged <30 years, it was observed that the oral progesterone group had higher rates compared to those in the IM progesterone group and in the vaginal progesterone gel group. When the pregnancy rates were compared in the group aged >35 years, the pregnancy rate in the group using oral progesterone was found to be higher than the pregnancy rate in the group using vaginal progesterone gel. In our study, only clinical pregnancy was evaluated, and the live birth rate was not. This is a limitation of our study.

SC administration is a relatively new progesterone administration route that we did not apply in our study but is one which has been compared to other methods in the literature. Türkgeldi et al. compared the pregnancy outcomes of females, who had received 50 mg of SC progesterone and 180 mg/day of vaginal progesterone in females who had undergone FET [19]. No statistically significant difference was observed between the pregnancy rate, live birth rate, and miscarriage rate. In their study, Venturella et al. administered SC progesterone to females, who had previously received vaginal progesterone [20]. The surveys conducted revealed that it is a simple and comfortable method that females are quite satisfied with. Our study did not evaluate the SC progesterone application, and we intend to conduct a similar study as a randomized controlled trial in the future.

We found that IM progesterone application was more effective than vaginal progesterone gel application for luteal phase support. Many randomized controlled, especially live birth rate studies, are required for the results to more closely approximate those for the general population. We plan to conduct randomized controlled trials encompassing the use of SC progesterone agents in the following stages.

## Figures and Tables

**Table 1:** Demographic data and treatment results of the females participating in the study.

	*n*	(%)
Treatment agent		
Crinone	57	34.8
Duphaston	30	18.3
Progestan	77	47
Age groups		
<30	41	25
30-35	59	36
>35	64	39
The cause of infertility		
Male	46	28
Female	75	45.7
Male and female	25	15.2
Unknown	18	11
Pregnancy		
No	72	43.9
Yes	92	56.1

	Mean (SD)	Median (min-Q1-Q3-max)
Age	33.24 (5.64)	33 (20-29.5-38-45)
BMI	25.15 (4.49)	24.23 (17.3-22-27.5-40)
FSH	8.28 (3.44)	7.43 (2.35-6.32-9.72-23.77)
LH	5.89 (2.64)	5.46 (0.42-3.95-7.56-14.9)
AMH	3.15 (3.18)	1.96 (0.01-0.96-4.58-15)
TSH	2.08 (1.08)	1.87 (0.12-1.41-2.55-6.03)
Basal E2	43.2 (14.71)	41.23 (12-32.1-50.3-97.27)
Transfer E2	396.27 (202.2)	354.5 (105.4-233.71-527.46-979)
Difference E2 (transfer-basal)	353.08 (201.64)	310.9 (44.59-193.74-489.33-951.1)
Basal progesterone	0.66 (0.31)	0.63 (0.14-0.42-0.86-1.76)
Transfer progesterone	20.36 (17.48)	14.35 (1.28-8.65-23-80)
The difference in progesterone (transfer-basal)	19.7 (17.5)	13.72 (0.8-8.02-22.03-78.69)
Endometrium (mm)	10.55 (2.16)	10.2 (7-9-12-22)
Number of attempts	1.7 (1.14)	1 (1-1-2-8)

SD.: standard deviation; min: minimum; Q1: percentile 25; Q3: percentile 75; max: maximum.

**Table 2:** Different treatment groups' demographic data and treatment results.

	All age groups				*p*
	Total	Vaginal P4 gel	Oral P4 tb	P4 IM	
	(*n* = 164)	(*n* = 57)	(*n* = 30)	(*n* = 77)	
Age (year) _median (min/max)_	33 (20/45)	31 (22/45)	34 (26/41)	33 (20/44)	0.287^k^
BMI (kg/m^2^) _median (min/max)_	24.23 (17.3/40)	23.55 (17.3/40)	23.55 (19.5/29.3)	25 (18/38.25)	0.234^k^
The cause of infertility _*n*__(%)_					0.245^ff^
Male	46 (28)	14 (24.6)	10 (33.3)	22 (28.6)	
Female	75 (45.7)	30 (52.6)	10 (33.3)	35 (45.5)	
Male and female	25 (15.2)	5 (8.8)	5 (16.7)	15 (19.5)	
Unknown	18 (11)	8 (14)	5 (16.7)	5 (6.5)	
Pregnancy _*n*__(%)_	92 (56.1)	24 (42.1)	17 (56.7)	51 (66.2)^a^	**0.021** ^**c**^
Endometrium (mm) _median (min/max)_	10.2 (7/22)	10 (7/15.4)	11.1 (8/14)	10 (7/22)	0.174^k^
Number of attempts (*n*) _median (min/max)_	1 (1/8)	1 (1/8)	1 (1/4)	1 (1/6)	0.635^k^
FSH (mlU/ml) _median (min_/_max)_	7.43 (2.35/23.77)	7.49 (2.69/22.6)	7.3 (4.61/18.2)	6.94 (2.35/23.77)	0.671^k^
LH(IU/l) _median (min/max)_	5.46 (0.42/14.9)	5 (1.12/13.52)^b^	6.72 (0.51/14.9)	5.21 (0.42/12.6)^b^	**0.001** ^**k**^
AMH (ng/ml) _median (min_/_max)_	1.96 (0.01/15)	1.9 (0.14/12.66)	1.96 (0.07/15)	2.05 (0.01/12.95)	0.907^k^
TSH (milliliter) _median (min/max)_	1.87 (0.12/6.03)	1.88 (0.3/6.03)	1.86 (0.19/3.26)	1.9 (0.12/4.74)	0.785^k^
E2 (pg/ml) _median (min/max)_					
Basal	41.23 (12/97.27)	41.2 (12/97.27)	45.15 (24.5/86.7)	39.59 (12.68/79)	0.129^k^
Transfer	354.5 (105.4/979)	354 (105.4/879.62)	353 (145/768)	356 (115/979)	0.827^k^
Difference (transfer-basal)	310.9 (44.59/951.1)	315.6 (68.66/851.62)	296.95 (104.2/717.2)	318.5 (44.59/951.1)	0.817^k^
*p* value for basal vs. transfer	**<0.001ᵚ**	**<0.001ᵚ**	**<0.001ᵚ**	**<0.001ᵚ**	
Progesterone (ng/ml) _median (min/max)_					
Basal	0.63 (0.14/1.76)	0.53 (0.14/1.51)	0.73 (0.26/1.04)	0.64 (0.21/1.76)	0.056^k^
Transfer	14.35 (1.28/80)	9.56 (3.13/78)	16.05 (5.4/60)	16.12 (1.28/80)	0.107^k^
Difference (transfer-basal)	13.72 (0.8/78.69)	9.22 (2.49/77.7)	15.27 (4.84/59.24)	15.68 (0.8/78.69)	0.119^k^
*p* value for basal vs. transfer	**<0.001ᵚ**	**<0.001ᵚ**	**<0.001ᵚ**	**<0.001ᵚ**	

^k^Kruskal-Wallis H test (Monte Carlo); Post hoc test: Dunn's test; ^ff^Fisher-Freeman-Halton test (Monte Carlo); ^c^Pearson's chi-square test (Monte Carlo); ^ᵚ^Wilcoxon test (Monte Carlo); ^a^expresses significance according to Crinone, ^b^expresses significance according to Duphaston; min: minimum; max: maximum.

**Table 3:** Demographic data and treatment results for females <30 years of age using different luteal phase support agents.

	Age group (<30)				*p*
	Total	Vaginal P4 gel	Oral P4 tb	P4 IM	
	(*n* = 41)	(*n* = 18)	(*n* = 6)	(*n* = 17)	
Age (year) _median (min/max)_	27 (20/29)	26 (22/29)	27.5 (26/29)	27 (20/29)	0.328^k^
BMI (kg/m^2^) _median (min/max)_	23.5 (17.3/38.06)	22.35 (17.3/31.11)	24.97 (21.8/26.5)	25 (18.75/38.06)	0.166^k^
The cause of infertility, _*n*__(%)_					0.460^f^^f^
Male	14 (34.1)	6 (33.3)	3 (50)	5 (29.4)	
Female	16 (39)	5 (27.8)	2 (33.3)	9 (52.9)	
Male and female	5 (12.2)	2 (11.1)	1 (16.7)	2 (11.8)	
Unknown	6 (14.6)	5 (27.8)	0 (0)	1 (5.9)	
Pregnancy _*n*__(%)_	26 (63.4)	9 (50)	2 (33.3)	15 (88.2)^a b^	**0.013^f^^f^**
Endometrium (mm) _median (min/max)_	11 (7.7/15.2)	10.05 (8.5/15.2)	11.5 (8/14)	11 (7.7/15)	0.545^k^
Number of attempts (*n*) _median (min/max)_	1 (1/3)	1 (1/3)	1 (1/3)	1 (1/3)	0.910^k^
FSH (mlU/ml) _median (min/max)_	6.9 (2.35/21)	7.18 (2.69/14.2)	6.65 (5.5/10.3)	6.72 (2.35/21)	0.964^k^
LH (IU/l) _median (min/max)_	5.39 (2.18/12.6)	5.1 (3.15/9.43)	7.94 (6.31/11.3)	5.02 (2.18/12.6)	0.055^k^
AMH (ng/ml) _median (min/max)_	4.6 (0.2/15)	3.67 (0.36/12.66)	2.47 (0.83/15)	5.08 (0.2/11.6)	0.520^k^
TSH (milliliter) _median (min/max)_	1.82 (0.19/6.03)	1.92 (0.93/6.03)	1.86 (0.19/2.44)	1.81 (0.84/3.98)	0.565^k^
E2 (pg/ml) _median (min/max)_					
Basal	40.8 (12/68)	41.15 (12/68)	44.3 (32/52.4)	38 (24.35/59)	0.158^k^
Transfer	334.84 (109.76/974.49)	344.92 (109.76/879.62)	243.5 (145/420)	404 (115/974.49)	0.363^k^
Difference (transfer-basal)	305.61 (68.66/934.15)	309.81 (68.66/851.62)	194.8 (104.2/371.4)	362 (83/934.15)	0.284^k^
*p* value for basal vs. transfer	**<0.001ᵚ**	**<0.001ᵚ**	**0.031ᵚ**	**<0.001ᵚ**	
Progesterone (ng/ml) _median (min/max)_					
Basal	0.69 (0.21/1.31)	0.67 (0.26/1.28)	0.77 (0.64/0.96)	0.66 (0.21/1.31)	0.608^k^
Transfer	12.8 (3.47/60)	9.55 (3.47/60)	15.55 (8.5/23.9)	15 (4.91/46.54)	0.377^k^
Difference (transfer-basal)	12.29 (2.78/59.6)	8.91 (2.78/59.6)	14.66 (7.78/23.26)	14.18 (4.25/46.08)	0.427^k^
*p* value for basal vs. transfer	**<0.001ᵚ**	**<0.001ᵚ**	**0.031ᵚ**	**<0.001ᵚ**	

^k^Kruskal-Wallis H test (Monte Carlo); ^ff^Fisher-Freeman-Halton test (Monte Carlo); ^ᵚ^Wilcoxon test (Monte Carlo); ^a^expresses significance according to Crinone; ^b^expresses significance according to Duphaston; min: minimum; max: maximum.

**Table 4:** Demographic data and treatment outcomes for different luteal phase support agents for females aged 30-35.

	Age group (30-35)				*p*
	Total	Vaginal P4 gel	Oral P4 tb	P4 IM	
	(*n* = 59)	(*n* = 19)	(*n* = 11)	(*n* = 29)	
Age (year) _median (min/max)_	32 (30/34)	31 (30/34)	32 (31/34)^a^	32 (30/34)	**0.021^k^**
BMI (kg/m^2^) _median (min/max)_	23.5 (18/33.33)	23.55 (18/33.33)	22.7 (19.5/27.8)	23 (18/33.2)	0.587^k^
The cause of infertility, _*n*__(%)_					0.068^f^^f^
Male	20 (33.9)	5 (26.3)	5 (45.5)	10 (34.5)	
Female	20 (33.9)	9 (47.4)	1 (9.1)	10 (34.5)	
Male and female	12 (20.3)	3 (15.8)	1 (9.1)	8 (27.6)	
Unknown	7 (11.9)	2 (10.5)	4 (36.4)	1 (3.4)	
Pregnancy _*n*__(%)_	38 (64.4)	11 (57.9)	9 (81.8)	18 (62.1)	0.449 ^f^^f^
Endometrium (mm) _median (min_/_max)_	10.5 (7/22)	10 (7/15.4)	11.2 (9/14)	9.8 (7.4/22)	0.181 ^k^
Number of attempts (*n*) _median (min/max)_	1 (1/6)	1 (1/6)	2 (1/4)	1 (1/6)	0.595 ^k^
FSH (mlU/ml) _median (min_/_max)_	7.34 (3.05/22.6)	7.34 (4.06/22.6)	6.7 (4.61/13.6)	7.5 (3.05/14.11)	0.290 ^k^
LH(IU/l) _median (min/max)_	5.64 (0.42/14.9)	5.78 (1.12/13.52)	5.84 (4.88/14.9)	5.45 (0.42/9.32)	0.141 ^k^
AMH (ng/ml) _median (min_/_max)_	2.3 (0.13/12.95)	1.9 (0.14/11.26)	3.5 (1.12/11.2)	2.05 (0.13/12.95)	0.141 ^k^
TSH (milliliter) _mean (SD)_	2.14 (1.01)	2.37 (1.22)	1.90 (0.83)	2.07 (0.92)	0.412 ^f^
E2 (pg/ml) _median (min/max)_					
Basal	44 (24.5/97.27)	44 (26/97.27)	45.3 (24.5/86.7)	40.1 (24.95/79)	0.699^k^
Transfer	394.1 (115/846)	394.1 (115/846)	456 (191/768)	374 (123.59/697.58)	0.354^k^
Difference (transfer-basal)	349.5 (44.59/793.25)	368.1 (71/793.25)	410.7 (147/717.2)	333.9 (44.59/643.58)	0.369^k^
*p* value for basal vs. transfer	**<0.001ᵚ**	**<0.001ᵚ**	**<0.001ᵚ**	**<0.001ᵚ**	
Progesterone (ng/ml) _median (min/max)_					
Basal	0.63 (0.24/1.33)	0.52 (0.24/1.16)	0.68 (0.35/0.92)	0.76 (0.3/1.33)^a^	**0.023** ^**k**^
Transfer	16.12 (2.42/80)	20.64 (3.9/74.49)	13.6 (5.4/56.5)	16.12 (2.42/80)	0.817^k^
Difference (transfer-basal)	15.4 (2.02/78.69)	19.85 (3.26/73.95)	13.25 (4.84/55.75)	15.4 (2.02/78.69)	0.766^k^
*p* value for basal vs. transfer	**<0.001ᵚ**	**<0.001ᵚ**	**<0.001ᵚ**	**<0.001ᵚ**	

^k^Kruskal-Wallis H test (Monte Carlo); Post hoc test: Dunn's test; ^ff^Fisher-Freeman-Halton test (Monte Carlo); ^f^One way ANOVA test (Boostrap); ^ᵚ^Wilcoxon test (Monte Carlo); ^a^expresses significance according to Crinone; SD: standard deviation; min: minimum; max: maximum.

**Table 5:** Demographic data and treatment outcomes for females aged >35 years for different luteal phase support agents.

	Age group (>35)				*p*
	Total	Vaginal P4 gel	Oral P4 tb	P4 IM	
	(*n* = 64)	(*n* = 20)	(*n* = 13)	(*n* = 31)	
Age _median (min/max)_	39 (35/45)	40 (35/45)	38 (35/41)	39 (35/44)	0.270^k^
BMI _median (min_/_max)_	25.38 (19.5/40)	24.78 (21/40)	24.5 (19.5/29.3)	26 (19.72/38.25)	0.211^k^
The cause of infertility _*n*__(%)_					0.302^f^^f^
Male	12 (18.8)	3 (15)	2 (15.4)	7 (22.6)	
Female	39 (60.9)	16 (80)	7 (53.8)	16 (51.6)	
Male and female	8 (12.5)	0 (0)	3 (23.1)	5 (16.1)	
Unknown	5 (7.8)	1 (5)	1 (7.7)	3 (9.7)	
Pregnancy _*n*__(%)_	28 (43.8)	4 (20)	6 (46.2)	18 (58.1)^a^	**0.031** ^**c**^
Endometrium (mm) _median (min_/_max)_	9.8 (7/14)	9.75 (7/12.9)	10 (8/13)	9.5 (7/14)	0.715^k^
Number of attempts (n) _median (min/max)_	1 (1/8)	1 (1/8)	1 (1/4)	2 (1/6)	0.850^k^
FSH (mlU/ml) _median (min_/_max)_	7.96 (3.84/23.77)	9.12 (4.19/19.44)	8.4 (4.73/18.2)	6.86 (3.84/23.77)	0.221^k^
LH(IU/L) _median (min/max)_	4.97 (0.51/12.24)	4.27 (2.43/10)	6.87 (0.51/10.7)	4.66 (0.96/12.24)	0.079^k^
AMH (ng/ml) _median (min_/_max)_	1.25 (0.01/11.88)	1.3 (0.15/8)	1.1 (0.07/6.5)	1.2 (0.01/11.88)	0.919^k^
TSH (milliliter) _mean (SD)_	1.95 (0.89)	1.77 (0.84)	1.97 (0.73)	2.06 (0.98)	0.484^f^
E2 (pg/ml) _median (min/max)_					
Basal	41.48 (12.68/85)	35.79 (21.99/81)	45.3 (31.4/85)	40.13 (12.68/76.93)	0.262^k^
Transfer	344.5 (105.4/979)	337.5 (105.4/829)	350 (231/643)	344 (134.1/979)	0.791^k^
Difference (transfer-basal)	300.52 (69.62/951.1)	307.16 (69.62/748)	295.9 (182.4/592.9)	304.87 (110.9/951.1)	0.807^k^
*p* value for basal vs. transfer	**<0.001ᵚ**	**<0.001ᵚ**	**<0.001ᵚ**	**<0.001ᵚ**	
Progesterone (ng/ml) _median (min/max)_					
Basal	0.55 (0.14/1.76)	0.52 (0.14/1.51)	0.76 (0.26/1.04)	0.48 (0.21/1.76)	0.129^k^
Transfer	15 (1.28/78)	9.47 (3.13/78)	16.8 (7.8/60)^a^	18 (1.28/60)^a^	**0.009^k^**
Difference (transfer-basal)	14.46 (0.8/77.7)	8.86 (2.49/77.7)	15.93 (7.04/59.24)^a^	17.3 (0.8/59.12)	**0.010^k^**
p value for basal vs. transfer	**<0.001ᵚ**	**<0.001ᵚ**	**<0.001ᵚ**	**<0.001ᵚ**	

^k^Kruskal Wallis H test (Monte Carlo), Post Hoc test: Dunn's test, ^ff^Fisher Freeman Halton test (Monte Carlo), ^f^One-way ANOVA test (Boostrap), ^c^Pearson Chi-Square test (Monte Carlo), ^ᵚ^Wilcoxon test (Monte Carlo), ^a^expresses significance according to Crinone, SD: standart deviation; min: minimum; max: maximum.

**Table 6:** The relationship between pregnancy results and age, treatment agents, FSH, *p* values on the day of transfer and pregnancy.

Reference groups: Pregnancy	B (SE.)	*p* value
Age (↓)	0.08 (0.03)	**0.017**
Treatment agent		
Vaginal P4 gel-oral P4 tb^R^	-0.75 (0.51)	0.142
Vaginal P4 gel-P4 IM^R^ (↑)	-1.3 (0.42)	**0.002**
Oral P4 tb-P4 IM^R^	-0.55 (0.47)	0.24
FSH (↓)	0.11 (0.05)	**0.038**
Transfer progesterone (↑)	0.05 (0.01)	**<0.001**
Constant	3.48 (1.22)	**0.004**
Accuracy rates	**No pregnancy: 48.6**	**Pregnancy: 88.0**

**Table 7:** Analysis of variables according to age groups.

Reference groups: pregnancy	<30 age		(30-35) age		>35 age		All patients	
	*p*	Odd ratio (95% C.I.)	*p*	Odd ratio (95% C.I.)	*p*	Odd ratio (95% C.I.)	*p*	Odd ratio (95% C.I.)
Treatment agents								
Oral P4 tb vs. vaginal P4 gel	0.163	25.35 (0.27-2,379.51)	0.39	2.92 (0.25-33.52)	0.15	4.02 (0.60-26.76)	0.214	2.02 (0.67-6.14)
P4 IM vs vaginal P4 gel	0.078	36.18 (0.67-1,958.33)	0.298	2.34 (0.47-11.66)	**P** ≤ 0.011	7.29 (1.58-33.58)	**0.004**	3.41 (1.49-7.78)
P4 IM vs. ral P4 tb	**0.041**	939.2 (1.33-664487.9)	0.834	1.27 (0.13-12.32)	0.564	1.57 (0.34-7.32)	0.32	1.66 (0.61-4.49)
Progesterone difference (ng/ml) (transfer-basal) (↑)	0.082	1.27 (0.98-1.55)	**0.023**	1.06 (1.01-1.11)	0.19	1.03 (0.99-1.07)	**0.001**	1.05 (1.02-1.07)
Accuracy rate	96.2/86.7/92.7		84.2/57.1/74.6		78.6/72.2/75.0		77.2/61.1/70.1	
Pregnancy/no pregnancy/general								

Multiple logistic regression (method = enter), C.I.: confidence interval.

## Data Availability

Requests for data will be considered by the corresponding author.
